# A green electrolysis of silver-decorated MoS_2_ nanocomposite with an enhanced antibacterial effect and low cytotoxicity†

**DOI:** 10.1039/d1na00100k

**Published:** 2021-03-29

**Authors:** Qilan Xu, Yuhui Liu, Ling Cai, Yue Cao, Feng Chen, Liuzhu Zhou, Ping Zhu, Huijun Jiang, Qiao-Yan Jiang, Yang Sun, Jin Chen

**Affiliations:** Center for Global Health, The Key Laboratory of Modern Toxicology, Ministry of Education, School of Public Health, Nanjing Medical University Nanjing 211166 China okachen30@gmail.com jchen@njmu.edu.cn; State Key Laboratory of Nuclear Resources and Environment, School of Nuclear Science and Engineering, East China University of Technology Nanchang 330013 China; Department of Forensic Medicine, Nanjing Medical University Nanjing 211166 China; School of Pharmacy, Nanjing Medical University Nanjing 211166 China; Jiangsu Province Engineering Research Center of Antibody Drug, Key Laboratory of Antibody Technique of National Health Commission, Nanjing Medical University Nanjing 211166 China

## Abstract

To tackle the devastating microbial infections for the public health, a continuous search for effective and safe nanobiocides based on their prominent nanoscale effects has been extensively explored during past decades. In this study, a green electrolysis method was employed to synthesize silver-doped molybdenum sulfide (Ag@MoS_2_) composite materials. The obtained nanocomposites exhibited a sheet-like structure with a large specific surface area, which contributed to the efficient loading and refined distribution of silver nanoparticles. G^−^*E. coli* and *G*^*+*^*S. aureus* were used as model bacteria for the antibacterial test, which revealed enhanced antibacterial activity of produced nanocomposites with an identified destructive effect on preformed biofilms. It was found that within 72 hour incubation, 20 μg mL^−1^ Ag@MoS_2_ was sufficient to inhibit the growth of *E. coli* and *S. aureus* without visible colony formation, pointing to a desirable long-term antibacterial activity. Further a mechanistic antibiosis study of Ag@MoS_2_ indicated the involvement of a generation of reactive oxygen species. Notably, owing to the well-distributed silver nanoparticles on the nontoxic MoS_2_ nanosheet, the cytotoxicity evaluation results revealed that produced nanocomposites exhibited negligible toxicity to mammalian cells, and thereby held promising potential for biomedical applications.

## Abstract 2

Silver-doped molybdenum sulfide (Ag@MoS_2_) nanocomposites with enhanced antibacterial activity and low toxicity.

## Graphical abstract

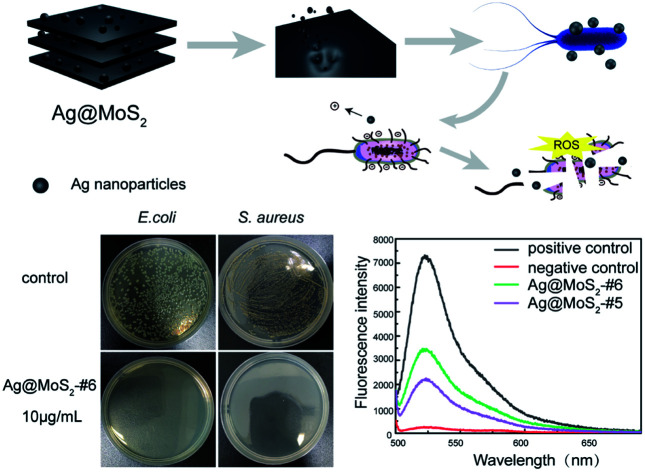

## 1. Introduction

Microbial infections have been considered a global issue threatening the public health, and is exacerbated by the emerging drug resistance due to the antibiotic misuse and inadvertent discharge.^1,2^ To solve this problem, it is urgent to develop safe and efficient antibacterial agents. The utilization of nanomaterials has shown promising potential to alleviate and/or combat microbial resistance.^3–5^ For example, a bunch of antibacterial nanomaterials, including graphene,^6^ carbon nanotubes,^7^ and metal oxide nanoparticles,^8^ have attracted increasing attention due to their superior antibacterial effect to overcome the existing drug resistance mechanism.^3^ In particular, silver-containing nanoparticles with a broad-spectrum antibacterial and low bacterial resistance, as well as antiviral effects, have been extensively used.^9^ Nevertheless, the excessive use of silver nanomaterials and their inherent aggregation-propensity at the nanoscale may raise safety concerns associated with some diseases, including spasms and gastrointestinal diseases.^10,11^ To achieve a good distribution for improved functioning and bioavailability, packaging silver nanoparticles in different nanocomposites is of practical importance.^12^

Molybdenum disulfide (MoS_2_), as a two-dimensional (2D) transition metal disulfide such as graphene,^13^ has been widely used in many fields, such as biomedicine,^14–17^ tribological,^18^ and optoelectronics^19^ due to its large specific surface area and excellent catalytic activity. In addition, Mo and S are essential trace elements for maintaining the cell physiological activities supporting the ideal candidate of MoS_2_ for vast biomedical applications.^20^ In particular, it was found that MoS_2_ of few-layered nanosheets possessed superior properties with the multilayered ones. For example, a vertically aligned MoS_2_ composed of a few layers exhibited excellent visible-light photocatalytic performance applicable for fast water disinfection.^21^

Among various synthesis strategies, the preparation of MoS_2_ nanomaterials by molybdenum salt electrolysis possesses unique advantages, such as eco-friendliness, low cost, and controllable morphology of the product,^16,18^ which led to the formation of MoS_2_ of distinct nanostructure correlating with its altered antibacterial effect.^22^ According to previous reports, 2D nanomaterials like graphene-supported silver nanoparticles can effectively reduce their agglomeration and alleviate drug resistance.^23–25^ As MoS_2_ and silver nanomaterials exert an antibacterial effect using different mechanisms, it is worthy of exploring the packaged Ag-doped MoS_2_ (Ag@MoS_2_) nanocomposite to maximize the bactericidal effect.

**Fig. 1:**
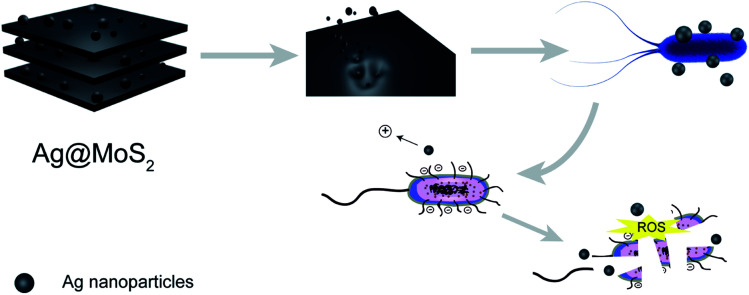
Preparation of the Ag@MoS_2_ nanocomposite with enhanced antibacterial effects.

In this study, we set out to synthesize the Ag@MoS_2_ nanocomposite of altered Ag content as a highly effective biocide using a green molten salt electrolysis method. The representative Gram-negative *E. coli* and Gram-positive *S. aureus* were used as models to test the antibacterial effects of the obtained nanocomposite, which indicated the correlation of the silver content of the nanocomposites with their antibacterial effect. As shown in Fig. 1, the antibiosis mechanistic study, antibacterial assay and cytotoxicity evaluation of the nanocomposite were also performed and discussed.^26^

## 2. Experimental

### 2.1. Materials

Hydrogen peroxide and crystal violet were from Sinopharm Chemical Reagent. Phosphate-buffered saline (PBS), DNTB (5,5′-dithiol-bis-(2-nitrobenzoic acid), Ellman's reagent), and reduced glutathione were obtained from Aladdin Co. (Shanghai, China). Calcein-AM was from Thermo Fisher Scientific (USA). The Reactive Oxygen Species (ROS) Assay Kit was obtained from Beyotime (Shanghai, China), and the Cell Counting Kit-8 was from MedChemExpress (USA). All reagents were used as received without further purifications.

### 2.2. Preparation of Ag@MoS_2_

A mixture of 35 g LiCl and 35 g KCl (63.7 : 36.3 mol%) molten salt was dehydrated under vacuum for 4.0 h at 473.0 K, and then imported into an alumina crucible located in a cylindrical quartz cell in an electric furnace. A graphite rod (6.0 mm in diameter) was applied as the counter electrode. The electrode was smoothed using SiC abrasive paper. A molybdenum wire (1.0 mm in diameter) that was smoothed with SiC abrasive paper was used as the working electrode. Ag@MoS_2_ nanosheets were prepared by galvanostatic electrolysis carried out on Mo electrodes in LiCl (35.0 g)-KCl (35.0 g)-K_2_MoO_4_ (4.0 g)-K_2_S (6.0 g)-AgCl (0.2, 0.5 and 1.0 g) melt at 823.0 K and current densities of 1.55 A cm^−2^ for 1.0 h, which were named as Ag@MoS_2_#1, Ag@MoS_2_#3 and Ag@MoS_2_#5, respectively. Ag@MoS_2_ nanosheets were prepared by galvanostatic electrolysis carried out on Mo electrodes in LiCl (35.0 g)-KCl (35.0 g)-K_2_MoO_4_ (4.0 g)-K_2_S (6.0 g)-AgCl (0.2, 0.5 and 1.0 g) melt at 1073.0 K and current densities of 5.94 A cm^−2^ for 1.0 h, which was labeled as Ag@MoS_2_#2, Ag@MoS_2_#4 and Ag@MoS_2_#6, respectively. After electrolysis, the precipitates were cleaned with distilled water, and finally dried under vacuum at 50.0 °C for 2.0 h.

### 2.3. Characterization

The constant potential/current meter was used to provide the current required for the experiment. The samples' microscopic features were collected on a scanning electron microscope (SEM, Hitachi SU-1510, Japan, accelerating voltage, 15 kV). Transmission electron microscopy (TEM) images were taken on an electron microscope (JEOL-1010, Japan). High-resolution TEM images were obtained using an electron microscope (FEI Tecnai G2 F30, USA). Elemental distribution analysis was performed by an energy dispersive spectrometer. X-ray photoelectron spectroscopy (XPS, PHI 5000 Versa Probe) was employed to study the surface elementary composition of the produced materials. The nitrogen sorption experiments were conducted on a Micromeritics ASAP-2020 volumetric adsorption analyzer (USA), and the specific surface areas of the materials were calculated based on the Brunauer–Emmett–Teller (BET) theory. The sample XRD patterns were recorded on a Smartlab TM 9 kW (Rigaku Corporation, Tokyo, Japan) equipped with a rotating anode and Cu Kα radiation (*λ* = 0.154 nm). Fluorescence spectra were obtained on an F-4700 Fluorescence spectrophotometer (Hitachi, Japan). The images of the fluorescent signals were acquired on a fluorescence microscope (ZEISS MTB2004, Germany).

### 2.4. Antibacterial experiments

To examine the antimicrobial effect of Ag@MoS_2_, *E. coli* (ATCC 25922) and *S. aureus* (ATCC 6538) were chosen as model bacteria, which were cultured overnight at 37 °C in Luria–Bertani (LB) media. An aliquot containing 100 μL bacterial suspension was subcultured in 5 mL LB media until the OD_600_ reached 0.50 (10^8^ CFU mL^−1^).^1^ The bacterial cells were reaped, and then washed three times with pre-sterilized saline solution (0.85% NaCl). Then, the final products were resuspended in saline solution. A mixture containing 100 μL diluted bacteria (10^6^ CFU mL^−1^) and 900 μL saline solution with different concentrations of Ag@MoS_2_ were incubated for 3 hours in a shaker at 37 °C. Then, 100 μL of the mixture was spread on LB agar plates and cultured for 16 h at 37 °C. Bacteria incubated in the absence of nanomaterials were used as the control.

### 2.5. In *vitro* interaction of the Ag@MoS_2_ nanosheets with *E. coil*

The morphologies of the Ag@MoS_2_ nanosheets combined with *E. coil* were observed under TEM. Specifically, 10 μl of bacteria suspension with Ag@MoS_2_ was placed on 200 mesh copper mesh carbon support film (Beijing Zhongjingkeyi Technology Co., Ltd. China), After fixing for 4 min, the surface liquid was blotted with filter paper, and then dyed with 3% heavy acid. Finally, the sample was observed under TEM (JEOL JEM-1010) at an acceleration voltage of 80 kV.

### 2.6. ROS measurement

To evaluate the ROS generation, 2′,7′-dichlorofluorescin-diacetate (DCFH-DA) was used as the dye to monitor the fluorescence images of the nanocomposites incubated with cells. The formation of ROS can be measured by the oxidation of nonfluorescent DCFH to highly fluorescent 2′,7′-dichlorofluorescin (DCF). After the nanocomposite was incubated with *E. coli* for 2 hours, the cells were stained with 10 μM DCFH-DA for 25 minutes, and the bacteria producing fluorescence signals were photographed on a fluorescence microscope (ZEISS MTB2004, Germany).

The measurement of intracellular ROS production was performed by staining *E. coli* with DCFH-DA.^27^ 100 μL of the obtained bacterial suspensions were added to 900 μL saline solution containing Ag@MoS_2_ (5 μg mL^−1^), which was dispensed into a microcentrifuge tube, followed by incubation for 2 h at 37 °C. After a washing step with the saline solution, the cells were stained with 10 μM DCFH-DA at 37 °C for 30 min in the dark. To remove excess unreacted fluorescent probes, cells were washed with PBS at least three times. The fluorescence intensity was measured with an excitation filter at 485 nm and an emission filter at 530 nm in a fluorospectrophotometer. Unstained cells were used as the negative control. The maximum fluorescence intensity values were observed after incubation with Rosup as a positive control.

### 2.7. Ellman's assay

Ag@MoS_2_ nanosheets were dispersed evenly by ultrasound. The dispersion of the Ag@MoS_2_ nanosheets (225 μL at 10 μg mL^−1^, 20 μg mL^−1^, or 40 μg mL^−1^) in 50 mM bicarbonate buffer was added into 225 μL of 0.8 mM glutathione (GSH) to trigger oxidation. The mixture was incubated in the dark at a speed of 150 rpm for 2–6 h. After an incubation period of 2–6 h, 785 μL of Tris–HCl and 15 μL of DNTB were added to the mixtures to obtain a yellow product. Ag@MoS_2_ nanosheets were removed from the mixtures by centrifugation. 200 μL of the centrifuged solution was then added to the 96-well microplate, and the absorbance at 412 nm was recorded on a microplate spectrophotometer. All tests were prepared in triplicate. The mixture of 1 mM of H_2_O_2_ and 0.4 mM GSH served as the positive control, and the GSH solution alone was the negative control. The loss of GSH was calculated by the following formula: loss of GSH % = (absorbance of negative control - absorbance of the sample)/absorbance of the negative control × 100.

### 2.8. Biofilm assay

*S. aureus* was cultured in LB medium for 12 h at 37 °C, and then diluted to 1.0 × 10^6^ CFU mL^−1^ by LB liquid medium (containing 1% glucose). To form biofilms, 200 μL of *Staphylococcus aureus* suspension was added into a 96-well plate and incubated at 37 °C for 24 h. After the establishment of *S. aureus* biofilms, the supernatant was removed, and 200 μL of Ag@MoS_2_#5 or Ag@MoS_2_#6 aqueous dispersions with different concentrations (25, 50, 100, 200 and 500 μg mL^−1^) was added into each well. Only 200 μL of saline solution was used as the control. The samples were incubated overnight at 37 °C. After fixation with 100 μL paraformaldehyde for 10 minutes, the supernatant of each well was removed and stained with 50 μL crystal violet (0.2%) for 30 minutes. Then, the samples were washed 3 times with saline solution and imaged with a Nikon E200 microscope.

### 2.9 Real water sample test

Environmental water samples were also tested for evaluating the killing effect of the obtained nanomaterials. Briefly, the 0.5 L glass sampler was pre-sterilized, and then the samples of rivers and lakes were collected at a depth of 30 cm in the middle of the water body, respectively. The total colony counting of the environmental water samples was followed by GB/T 5750.12-2006 and GB/T 5750.2-2006. 500 μL of the environmental water samples were added to 500 μL saline solution containing different concentrations of Ag@MoS_2_#5 or Ag@MoS_2_#6. After incubation for 3 hours in a shaker at 37 °C, 100 μL of the mixture was spread on LB agar plates and then cultured for 12 h at 37 °C.

### 2.10. Ag^+^ release test

Samples (Ag@MoS_2_#1–6, *n* = 6) were dispersed in 3 mL of PBS (pH = 7.3) at 37 °C. At a certain time (8 or 24 h), the Ag^+^ release solution (1.5 mL) was taken out and an equivalent amount of PBS was supplemented. The releasing amount of Ag^+^ was determined by ICP-MS. (ICAP Q, USA).

### 2.11. Cytotoxicity assay

To measure the cytotoxicity of the produced nanocomposites on human bronchial epithelial cells (BEAS-2B), 4 × 10^3^ cells were seeded in a 96-well plate (*n* = 3), which were incubated for 24 h until the cells began to adhere. Then, the culture medium was removed and different concentrations of Ag@MoS_2_#5 or Ag@MoS_2_#6 were added and incubated for 24 h, respectively, followed by another 2 h-incubation with Cell Counting Kit-8 solution (CCK-8, 10 μL per well). The color change of the solution at the wavelength of 450 nm was measured on Varioskan Flash (TecanInfinite M200 Pro, Switzerland). The cell viability was expressed as a relative percentage compared to untreated cells. The cytotoxicity of MoS_2_#6 mixed with colloidal silver was also evaluated for comparison.

## 3. Results and discussion

### 3.1. Characterization of Ag@MoS_2_

Using Mo electrodes, the Ag@MoS_2_ nanocomposite was prepared by potentiostat electrolysis with different Ag content as listed in Table 1. In addition, the morphology of the product was observed by SEM. As shown in Fig. S1,† many uniform Ag@MoS_2_ nanosheets were obtained exhibiting smooth and thin morphology. The size range of the nanosheet was 50–100 nm. To further validate the formation of the nanosheets and Ag nanoparticles, TEM was used to record the morphologies of the obtained materials. As shown in Fig. S2,† a bunch of Ag@MoS_2_ nanosheets with a few thin layers were observable, which supported a good distribution of Ag nanoparticles of relatively small size.

**Table 1:** The XPS assay of the Ag content of the samples (%)

Samples	Ag Content
Ag@MoS_2_#1	1.5%
Ag@MoS_2_#2	2.15%
Ag@MoS_2_#3	2.44%
Ag@MoS_2_#4	2.56%
Ag@MoS_2_#5	3.26%
Ag@MoS_2_#6	6.72%

**Fig. 2:**
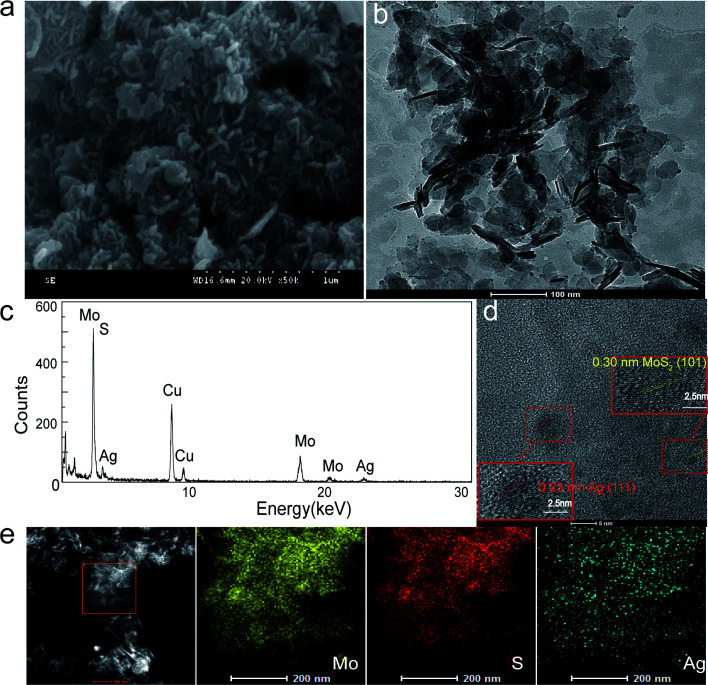
Morphological features of the Ag-doped MoS_2_#6 nanosheets. (a) SEM, (b) TEM, and (c) energy-dispersive X-ray spectrum. (d) HRTEM images with lattice spacing measurement. (e) The corresponding elemental mapping for Mo, S and Ag.

Due to the potent antibacterial activity of the silver nanoparticles, we chose the Ag-doped molybdenum sulfide with the highest Ag content for the following study. The representative SEM and HRTEM images of Ag@MoS_2_ nanosheets with a loading amount of 6.72% (Ag@MoS_2_#6) are shown in Fig. 2. It was evident that the Ag@MoS_2_ nanosheets exhibited a well-layered structure. The Ag nanoparticles adhered to the edges of the nanosheets, as shown in the HRTEM image, which confirmed the successful synthesis of the Ag@MoS_2_#6 nanosheets. As shown in Fig. 2d, the high-resolution TEM image of different samples showed a clear crystal lattice, revealing the good crystallization performance of the nanoparticles. Several selected regions of MoS_2_ and Ag were detected, and they revealed the lattice spacings of 0.23 nm (Ag (111))^28^ and 0.30 nm (MoS_2_ (101)),^18^ which was in agreement with the XRD results. Besides, the EDX element mapping analysis (Fig. 2c and e) illustrated the presence of Mo, S, and Ag elements, revealing the uniform deposition of Ag nanoparticles on the MoS_2_ nanosheets. The specific surface area of Ag@MoS_2_#3 was calculated to be 28.064 m^2^ g^−1^ based on the Brunauer–Emmett–Teller (BET) theory, and was found to be larger than that of MoS_2_ (9.17 m^2^ g^−1^). The material structure tended to have a larger specific surface area, which determined the high Ag doping and showed an advantage in the antibacterial effect.

**Fig. 3:**
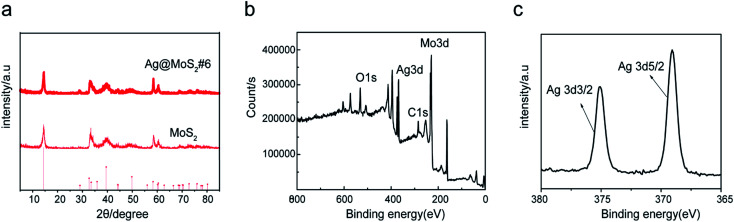
XRD patterns (a), XPS spectra of Ag@MoS_2_#6 (b), the XPS map of Ag3d elements (c).

To characterize the crystal structure of the materials, XRD tests of different products were carried out. Fig. 3a showed the XRD spectra of the prepared Ag@MoS_2_#6 nanosheets. The diffraction peaks at 14.5, 33.0, 39.8, 49.8, 58.4 and 60.4° were attributed to the (002), (101), (103), (105), (110) and (108) crystalline planes of MoS_2_, respectively, according to the MoS_2_ standard card (JCPDS no: 37-1492).^29,30^ No additional peaks were observed in the XRD diagram, indicating a good formation of the Ag nanoparticles-doped MoS_2_ nanosheets with high purity.

To further look at the chemical states of the surface elements in the Ag@MoS_2_ nanocomposites, the X-ray photoelectron spectroscopy (XPS) measurement of Ag@MoS_2_#6 nanosheets was carried out. The wide spectra of the Ag@MoS_2_#6 nanosheets shown in Fig. 3b demonstrated the presence of C, O, Mo, S, and Ag elements on the surface of the formed nanocomposite. Two well-defined peaks at 369.1 and 374.9 eV in the high-resolution spectrum of Ag 3d were attributed to Ag 3d_5/2_ and Ag 3d_3/2_,^5^ which were derived from the Ag nanoparticles (Fig. 3c). Taking these observations together, the Ag@MoS_2_ nanosheets were successfully formed through the electrolysis process.

### 3.2. Antibacterial activity

As the Ag nanoparticles normally exhibited wide-spectrum antimicrobial properties,^31^ we used two typical bacteria (including G^−^*E. coli* and G^+^*S. aureus*) as models in the assay. Fig. S3† presents the corresponding antibacterial models using agar plates of Ag@MoS_2_ nanosheets with Ag content ratios of 1.5%, 2.44%, 2.15%, 2.56%, 3.26%, and 6.72%, respectively. According to the results, in two typical bacterial samples, almost no colony formation was observed in 5 μg mL^−1^ Ag@MoS_2_ nanosheets with Ag content ratios of 6.72% (Ag@MoS_2_#6) and 3.26% (Ag@MoS_2_#5), which were consistent with the release tests in Table 2. The observation of *E. coli* being more susceptible to the obtained Ag@MoS_2_ may be attributed to the structural and compositional difference of the cell wall between *E. coli* (G^−^) and *S. aureus* (G^+^).^32^ For comparison, the antibacterial effect of MoS_2_, colloidal silver and MoS_2_#6 mixed with colloidal silver (same Ag concentration with that of the Ag@MoS_2_#6 group) against *E. coli* were also studied. As shown in Fig. S4,† the sample of MoS_2_ mixed with colloidal silver exhibited much less pronounced antibacterial ability towards *E. coli*, as compared with that of the Ag@MoS_2_#6 group over an incubation period up to 72 hours.

**Table 2:** The content of silver released at 8 h and 20 h measured by ICP-MS

Samples	Total (μg)	8 h (μg)	20 h (μg)
Ag@MoS_2_#1	3.47	0.18	1.31
Ag@MoS_2_#2	8.02	0.48	4.09
Ag@MoS_2_#3	6.02	0.7	4.32
Ag@MoS_2_#4	7.63	0.90	2.89
Ag@MoS_2_#5	6.62	0.99	4.15
Ag@MoS_2_#6	15.67	10.23	13.67

**Fig. 4:**
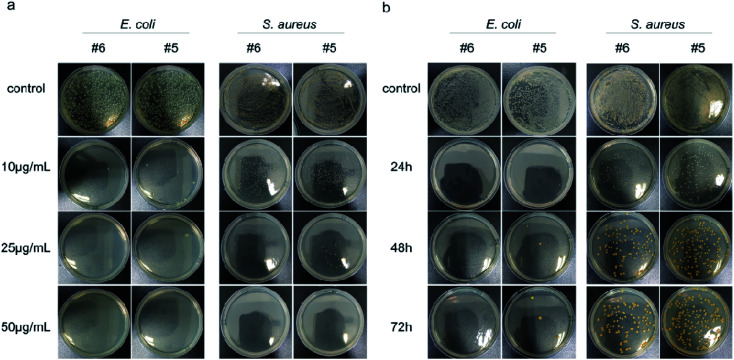
The optical images of the dose-dependent inhibition of the antibacterial ability (a) and long-term antibacterial effect (b) after incubation for 4 h with #6 (Ag@MoS_2_#6) and #5 (Ag@MoS_2_#5) of different concentrations.

Next, the dose-dependent inhibitory effects of two materials with better antibacterial properties (Ag@MoS_2_#5, Ag@MoS_2_#6) on the bacterial growth were assessed by a spread-plate method (Fig. 4a). Compared to the control group, Ag@MoS_2_#5 and Ag@MoS_2_#6 nanosheets exhibited a dose-dependent inhibitory effect on the growth of two typical bacteria. The antibacterial efficiency of the materials against *E. coli* calculated through manual counting (Table S1†) was almost 100% at the concentration of 10 μg mL^−1^, while the capability of Ag@MoS_2_#5 and Ag@MoS_2_#6 to kill *S. aureus* was 91.9% and 81.5%, respectively, which indicated that *E. coli* was more sensitive than *S. aureus*. Also, the presence of Ag@MoS_2_#5 and Ag@MoS_2_#6 (20 μg mL^−1^) resulted in almost no visible *E. coli* and *S. aureus* bacterial colonies within the incubation time of 72 h (Fig. 4b). We concluded that Ag@MoS_2_ possessed the prolonged effect of wide-spectrum antimicrobial activity, which was attributed to the refined microstructure of Ag@MoS_2_ being favorable for the controlled release of Ag nanoparticles.

**Fig. 5:**
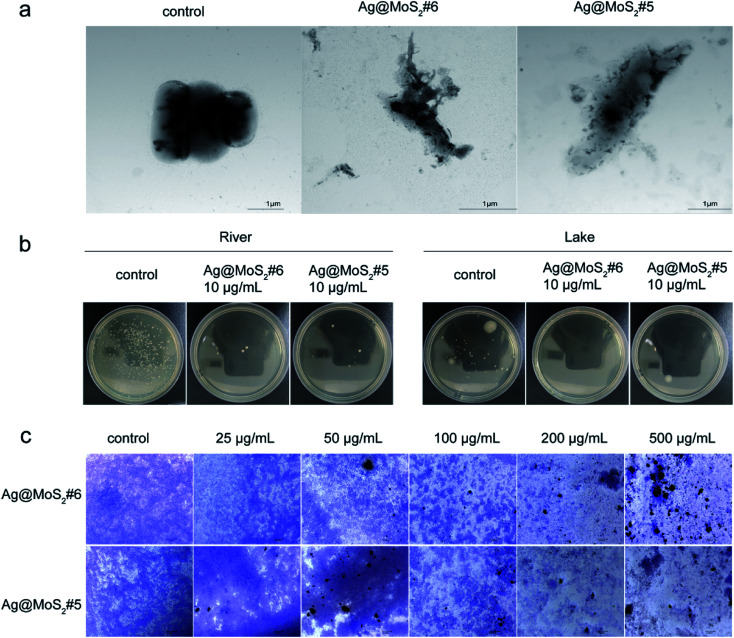
(a) Morphology change of *E. coli* after incubation with Ag@MoS_2_#6 or Ag@MoS_2_-#5, as observed by TEM. (b) Environmental water samples from the river and lake were treated with Ag@MoS_2_#6 or Ag@MoS_2_#5 at 10 μg mL^−1^. (c) Treatment of the established *S. aureus* biofilms by Ag@MoS_2_. Micrographs of crystal violet stained *S. aureus* biofilms treated with different concentrations of Ag@MoS_2_#6 or Ag@MoS_2_#5 (25, 50, 100, 200 and 500 μg mL^−1^).

To gain more information about the antibacterial properties of Ag@MoS_2_, the morphological changes of the bacteria cells in the presence of the formed nanomaterials were visualized by TEM. As shown in Fig. 5a, the untreated *E. coli* exhibited typical rod-shapes with a smooth surface. By contrast, in the samples treated with Ag@MoS_2_#5 or Ag@MoS_2_#6, it was found that the cell surface distortion of *E. coli* with evident leakage of cytoplasmic content happened during the incubation. This observation is due to the released Ag^+^ from the produced nanocomposite upon adherence to bacteria through electrostatic attraction, which leads to the apparent morphological changes and/or in worse case, the complete cell surface disruption.^33^

Given the increasing antimicrobial resistance in the surrounding environment, such as in water, it is highly urgent to explore the green biocide to tackle the bacterial contamination for the public health.^1^ As shown in Fig. 5b, according to the protocol of a national standard, the antibacterial effect of Ag@MoS_2_#6 on environmental water samples was tested. It was observed that the produced nanocomposites exhibited evident inhibitory effect against bacterial growth from both lake and river samples, which indicated the practical importance of silver-doped MoS_2_ towards microbial contaminant control in water.

### 3.3. *In vitro* treatment of biofilms

As the extracellular polymeric substances (EPS) of bacteria may form biofilms in response to the antibiotics functioning, it remains challenging to completely eliminate established biofilms in comparison with that of planktonic bacteria.^34,35^ To further evaluate the destructive effect of Ag@MoS_2_ on biofilms, the preformed *S. aureus* biofilms were treated with Ag@MoS_2_#5 and Ag@MoS_2_#6 at different concentrations, followed by the visualization based on the crystal violet staining.^26^ As shown in Fig. 5c, the micrographs of *S. aureus* biofilms were recorded. In the control group, a large number of live cells in violet were observed. By comparison, an evident concentration-dependent trend was identified when Ag@MoS_2_ was added, signifying the efficiency of the produced composite to kill bacterial cells by disrupting the formed biofilms. Meanwhile, at the same amount of incubated materials, a relative pronounced inhibitory effect of Ag@MoS_2_#6 on the biofilms was observed compared with that of Ag@MoS_2_#5. These results suggested that the obtained Ag@MoS_2_ possessed a promising advantage to tackle the microbial drug-resistance.

### 3.4. Antibacterial mechanism

Previous studies on MoS_2_ suggested that their antimicrobial properties involved the role of the ROS-independent mechanism, which resulted from disrupting the certain microbial membranes by nanomaterials.^22^ We thus conducted the Ellman's assay first to examine the oxidation capacity of produced MoS_2_ nanomaterials using GSH as an indicator of oxidative stress status.^36^ As shown in Fig. S5,† the GSH oxidation ratio by Ag@MoS_2_#6 reached only 7.5% (2 hour) and 20% (6 hour) at 40 μg mL^−1^. Such low oxidation capacity of the produced Ag@MoS_2_ toward GSH may argue against the possibilities of the ROS-independent pathway involved in the antibacterial process.

**Fig. 6:**
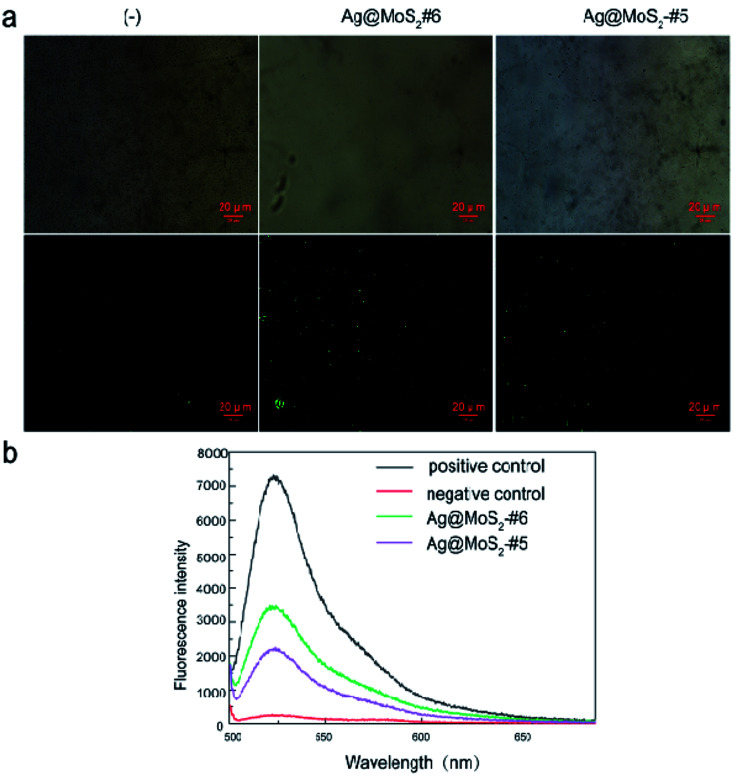
(a) Fluorescence microscopic images and bright field images of DCF^+^-labeled *E. coli* stained with DCFDA after the incubation with LB liquid media (negative control), Ag@MoS_2_#6 or Ag@MoS_2_#5. (b) The fluorescence intensity was measured with an excitation filter at 485 nm and an emission filter at 530 nm in a fluorospectrophotometer.

It was reported that nanoparticles (AgNPs) exerted antibacterial effects through two main mechanisms. On the one hand, the released silver ions (Ag^+^) from AgNPs may interact with cellular proteins and enzymes, causing serious structural deformation of the cell membrane,^37,38^ as reflected by the TEM (Fig. 5a), as well as the ICP-MS observations (Table 2). On the other hand, the generation of the reactive oxygen species (ROS) by AgNPs may perturb the cell metabolism.^39^ We thus further explored the underlying antibacterial mechanism of Ag@MoS_2_ by measuring the ROS production in the samples, in which the DCFH-DA probe was used to assess the generated ROS level induced by silver nanoparticles. *E. coli* was first incubated with Ag@MoS_2_ for 2 h, followed by 30 min staining with DCFH-DA, which could be oxidized by the ROS in the sample to generate fluorescent DCF. As shown in Fig. 6b, compared to the negative control, the fluorescence intensity of DCF in the solution reflecting the ROS level induced by 10 μg mL^−1^ Ag@MoS_2_ was increased significantly. Moreover, the ROS production of Ag@MoS_2_#6 was substantially higher than that of Ag@MoS_2_#5, correlating with the amount of silver content in the nanocomposite. Fluorescence microscopic images and bright field images of DCF-labeled *E. coli* are also shown in Fig. 6a. It was found that compared to that of Ag@MoS_2_#5, there was more green fluorescence in the recorded microscopic images of Ag@MoS_2_#6, which demonstrated that the antibacterial mechanism of the obtained nanocomposite may be related to the production of ROS.

### 3.5 Cytocompatibility evaluation

**Fig. 7:**
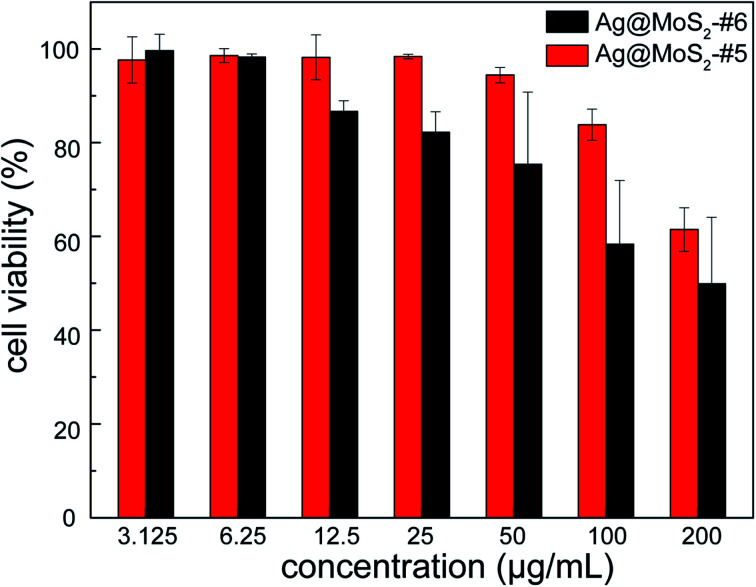
The cytotoxicity of Ag@MoS_2_#5 and Ag@MoS_2_#6 on BEAS-2B cells. Error bars represented the standard deviations (*n* = 3).

To evaluate the cytocompatibility of the obtained nanocomposite that is crucial for its practical utilization, human lung epithelial BEAS-2B cells were incubated in the presence of Ag@MoS_2_#5 and Ag@MoS_2_#6, followed by the measurement using CCK-8 on a microplate reader. As shown in Fig. 7, the evaluation results indicated that after 24 h incubation, Ag@MoS_2_ exhibited negligible toxicity to the tested mammalian cells even at the concentration of up to 100 μg mL^−1^. It should be noted that 10 μg mL^−1^ Ag@MoS_2_ was shown to be efficient to inhibit the growth of the tested bacterial cells (Fig. 4). Therefore, such satisfactory results of the cellular compatibility evaluation indicated that the obtained Ag@MoS_2_ is readily applicable for practical uses with ensured safety. Additionally, at the same concentration of Ag, the mixture of MoS_2_ and colloidal silver showed higher cytotoxicity than that of Ag@MoS_2_#6, suggesting an advantageous biomedical perspective of the obtained Ag@MoS_2_ (Fig. S6†).

## 4. Conclusion

In this study, we have successfully synthesized the Ag nanoparticles-loaded MoS_2_ nanosheets based on the green electrolysis method. Due to the 2D planar structure of the high specific surface area, the MoS_2_ nanosheets achieved the sufficient loading of Ag nanoparticles with good distribution, which was reflected by the visible crystal lattice space measurement. Moreover, compared with pure MoS_2_, the formed Ag@MoS_2_ exhibited relatively strong antibacterial activities against *E. coli* and *S. aureus*. In particular, two types of Ag@MoS_2_ nanocomposites of higher silver content were chosen for the antibacterial test, which demonstrated an evident long-term inhibitory effect on the bacterial cells growth and a disruptive morphological change of the preformed biofilms. Taking advantage of the high biocompatibility and remarkable bactericidal effect on the real water sample, the synthesized nanocomposites showed promising potential for environmental and hygienic uses. In the long run, due to its superior photoelectric and photothermal properties, further modular adaptions on Ag@MoS_2_ may provide alternative solutions towards emerging drug resistance in clinics, such as tumor chemotherapy.

## Author contributions

Qilan Xu, Ling Cai: methodology, experiment, writing – original draft. Ping Zhu, Liuzhu Zhou: methodology. Yuhui Liu, Yue Cao, Feng Chen, Qiao-Yan Jiang, Yang Sun, Huijun Jiang: methodology, writing – review & editing. Jin Chen: conceptualization, supervision, writing – review & editing.

## Conflicts of interest

The authors declare that there is no conflict of interest.

## Supplementary Material

NA-003-D1NA00100K-s001

## References

[cit1] Cabral J. P. S. (2010). Water Microbiology. Bacterial Pathogens and Water. Int. J. Environ. Res. Public Health.

[cit2] Yu S. (2018). *et al.*, Dendritic Fe_3_O_4_@Poly(dopamine)@PAMAM Nanocomposite as Controllable NO-Releasing Material: A Synergistic Photothermal and NO Antibacterial Study. Adv. Funct. Mater..

[cit3] Pelgrift R. Y., Friedman A. J. (2013). Nanotechnology as a therapeutic tool to combat microbial resistance. Adv. Drug Delivery Rev..

[cit4] Ding X. (2019). *et al.*, Biodegradable Antibacterial Polymeric Nanosystems: A New Hope to Cope with Multidrug-Resistant Bacteria. Small.

[cit5] Xie X. (2017). *et al.*, Synergistic Bacteria Killing through Photodynamic and Physical Actions of Graphene Oxide/Ag/Collagen Coating. ACS Appl. Mater. Interfaces.

[cit6] Wang R. (2020). *et al.*, Graphdiyne-modified TiO2 nanofibers with osteoinductive and enhanced photocatalytic antibacterial activities to prevent implant infection. Nat. Commun..

[cit7] Qiao Y. (2020). *et al.*, Treatment of MRSA-infected osteomyelitis using bacterial capturing, magnetically targeted composites with microwave-assisted bacterial killing. Nat. Commun..

[cit8] Pantaroto H. N. (2018). *et al.*, Antibacterial photocatalytic activity of different crystalline TiO2 phases in oral multispecies biofilm. Dent. Mater..

[cit9] Xiu Z.-m. (2012). *et al.*, Negligible Particle-Specific Antibacterial Activity of Silver Nanoparticles. Nano Lett..

[cit10] Kittler S. (2010). *et al.*, Toxicity of Silver Nanoparticles Increases during Storage Because of Slow Dissolution under Release of Silver Ions. Chem. Mater..

[cit11] Awasthi K. K. (2013). *et al.*, Silver nanoparticle induced cytotoxicity, oxidative stress, and DNA damage in CHO cells. J. Nanopart. Res..

[cit12] Song Y. (2018). *et al.*, Silver-Incorporated Mussel-Inspired Polydopamine Coatings on Mesoporous Silica as an Efficient Nanocatalyst and Antimicrobial Agent. ACS Appl. Mater. Interfaces.

[cit13] Tan C., Zhang H. (2015). Two-dimensional transition metal dichalcogenide nanosheet-based composites. Chem. Soc. Rev..

[cit14] Zhang W. (2016). *et al.*, Versatile molybdenum disulfide based antibacterial composites for in vitro enhanced sterilization and in vivo focal infection therapy. Nanoscale.

[cit15] Cao W., Yue L., Wang Z. (2019). High antibacterial activity of chitosan – molybdenum disulfide nanocomposite. Carbohydr. Polym..

[cit16] Yang A. (2020). *et al.*, Electrochemical generation of liquid and solid sulfur on two-dimensional layered materials with distinct areal capacities. Nat. Nanotechnol..

[cit17] Nguyen E. P., Castro Silva C. . d. C., Merkoci A. (2020). Recent advancement in biomedical applications on the surface of two-dimensional materials: from biosensing to tissue engineering. Nanoscale.

[cit18] Liu Y.-H. (2018). *et al.*, Electrochemical synthesis and tribological properties of flower-like and sheet-like MoS_2_ in LiCl KCl (NH4)_6_Mo_7_O_24_KSCN melt. Electrochim. Acta.

[cit19] Mazaheri A. (2020). *et al.*, MoS2-on-paper optoelectronics: drawing photodetectors with van der Waals semiconductors beyond graphite. Nanoscale.

[cit20] Kim J., Kim H., Kim W. J. (2016). Single-Layered MoS_2_-PEI-PEG Nanocomposite-Mediated Gene Delivery Controlled by Photo and Redox Stimuli. Small.

[cit21] Liu C. (2016). *et al.*, Rapid water disinfection using vertically aligned MoS2 nanofilms and visible light. Nat. Nanotechnol..

[cit22] Xu Q. (2020). *et al.*, Electrochemical formation of distinct nanostructured MoS_2_ with altered antibacterial activity. Mater. Lett..

[cit23] Zangeneh Kamali K. (2015). *et al.*, Silver@graphene oxide nanocomposite-based optical sensor platform for biomolecules. RSC Adv..

[cit24] Awasthi K. K. (2013). *et al.*, Silver nanoparticle induced cytotoxicity, oxidative stress, and DNA damage in CHO cells. J. Nanopart. Res..

[cit25] Tan S. (2020). *et al.*, Enhanced synergetic antibacterial activity by a reduce graphene oxide/Ag nanocomposite through the photothermal effect. Colloids Surf., B.

[cit26] Yuwen L. (2018). *et al.*, MoS2@polydopamine-Ag nanosheets with enhanced antibacterial activity for effective treatment of Staphylococcus aureus biofilms and wound infection. Nanoscale.

[cit27] Shekhova E., Kniemeyer O., Brakhage A. A. (2017). Induction of Mitochondrial Reactive Oxygen Species Production by Itraconazole, Terbinafine, and Amphotericin B as a Mode of Action against Aspergillus fumigatus. Antimicrob. Agents Chemother..

[cit28] Su D. S. (2008). *et al.*, Surface Chemistry of Ag Particles: Identification of Oxide Species by Aberration-Corrected TEM and by DFT Calculations. Angew. Chem..

[cit29] Maitra U. (2013). *et al.*, Highly Effective Visible-Light-Induced H2Generation by Single-Layer 1T-MoS2and a Nanocomposite of Few-Layer 2H-MoS2with Heavily Nitrogenated Graphene. Angew. Chem., Int. Ed..

[cit30] Ai X. (2018). *et al.*, XPS and Raman study of the active-sites on molybdenum disulfide nanopetals for photocatalytic removal of rhodamine B and doxycycline hydrochlride. RSC Adv..

[cit31] Lara H. H. (2011). *et al.*, Silver nanoparticles are broad-spectrum bactericidal and virucidal compounds. J. Nanobiotechnol..

[cit32] Ghodake G., Lim S.-R., Lee D. S. (2013). Casein hydrolytic peptides mediated green synthesis of antibacterial silver nanoparticles. Colloids Surf., B.

[cit33] Richter A. P. (2015). *et al.*, An environmentally benign antimicrobial nanoparticle based on a silver-infused lignin core. Nat. Nanotechnol..

[cit34] Costerton J. W., Stewart P. S., Greenberg E. P. (1999). Bacterial Biofilms: A Common Cause of Persistent Infections. Science.

[cit35] Fisher R. A., Gollan B., Helaine S. (2017). Persistent bacterial infections and persister cells. Nat. Rev. Microbiol..

[cit36] Yin W. (2016). *et al.*, Functionalized Nano-MoS2with Peroxidase Catalytic and Near-Infrared Photothermal Activities for Safe and Synergetic Wound Antibacterial Applications. ACS Nano.

[cit37] Rizzello L., Pompa P. P. (2014). Nanosilver-based antibacterial drugs and devices: Mechanisms, methodological drawbacks, and guidelines. Chem. Soc. Rev..

[cit38] Rai M., Yadav A., Gade A. (2009). Silver nanoparticles as a new generation of antimicrobials. Biotechnol. Adv..

[cit39] Choi O., Hu Z. (2008). Size Dependent and Reactive Oxygen Species Related Nanosilver Toxicity to Nitrifying Bacteria. Environ. Sci. Technol..

