# Supplementary material for: A green electrolysis of silver-decorated MoS2 nanocomposite with an enhanced antibacterial effect and low cytotoxicity
Source: Nanoscale Adv. 2021 Mar 29;3(12):3460–9. doi: 10.1039/d1na00100k (PMC9417968; doi:10.1039/d1na00100k)
Supplement: NA-003-D1NA00100K-s001 [file NA-003-D1NA00100K-s001.pdf]

Electronic Supplementary Material (ESI) for Nanoscale Advances.
This journal is © The Royal Society of Chemistry 2021

Supporting Information

Green electrolysis of silver decorated MoS_2 nanocomposite with enhanced antibacterial effect and low cytotoxicity

Qilan Xu[1#], Yuhui Liu[2#], Ling Cai[1#], Yue Cao[3], Feng Chen[3], Liuzhu Zhou[1], Ping Zhu[1], Huijun Jiang[4], Qiao-Yan Jiang[3], Yang Sun[3], Jin Chen[1,5*]

[1]Center for Global Health & The Key Laboratory of Modern Toxicology, Ministry of Education, School of Public Health, Nanjing Medical University, Nanjing 211166, China

[2]State Key Laboratory of Nuclear Resources and Environment, School of Nuclear Science and Engineering, East China University of Technology, Nanchang, 330013, China

[3]Department of Forensic Medicine, Nanjing Medical University, Nanjing, 211166, China

[4]School of Pharmacy, Nanjing Medical University, Nanjing 211166, China

[5]Jiangsu Province Engineering Research Center of Antibody Drug, Key Laboratory of Antibody Technique of National Health Commission, Nanjing Medical University, Nanjing 211166, China

[#]Equal contribution.

*Corresponding author. Email: okachen30@gmail.com; jchen@njmu.edu.cn

Table S1. Counting Results (CFU) of *E.coil* and *S.aureus* cultured with different concentrations of materials from Figure 4a

Group	*E. coli*		*S. aureus*	
	#6	#5	#6	#5
control	1024	1168	6500	4028
10 μg/mL	1	7	528	747
20 μg/mL	0	1	1	48
50 μg/mL	0	0	1	4

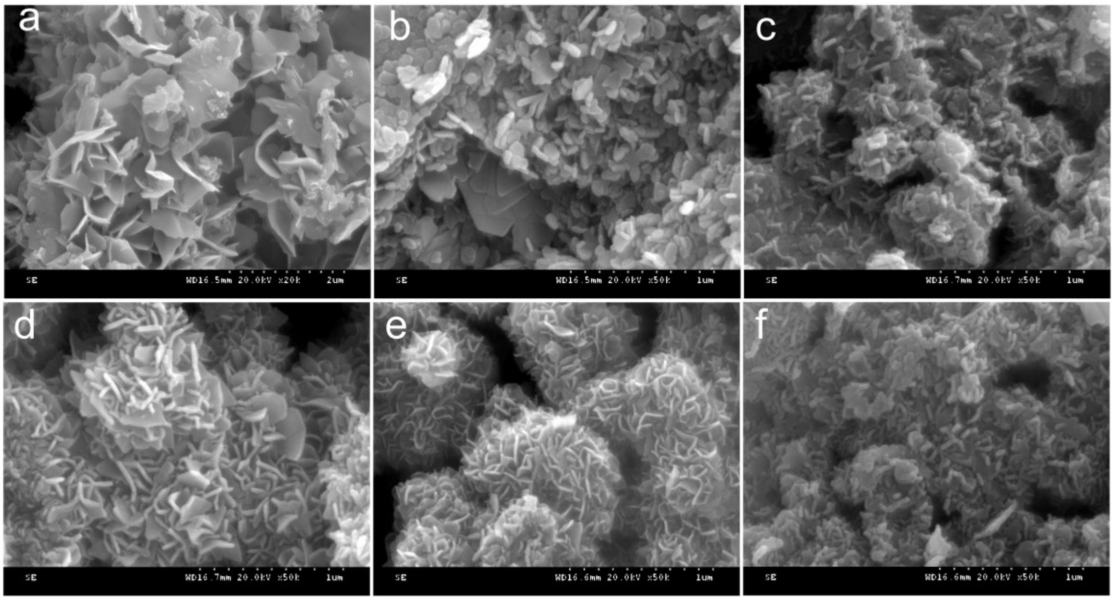

Figure S1. The SEM image of an Ag-doped MoS_2 nanosheets with the weight ratios of Ag of 1.5 % (a) , 2.15 % (b), 2.44 % (c), 2.56 % (d), 3.26 % (e), and 6.72 % (f).

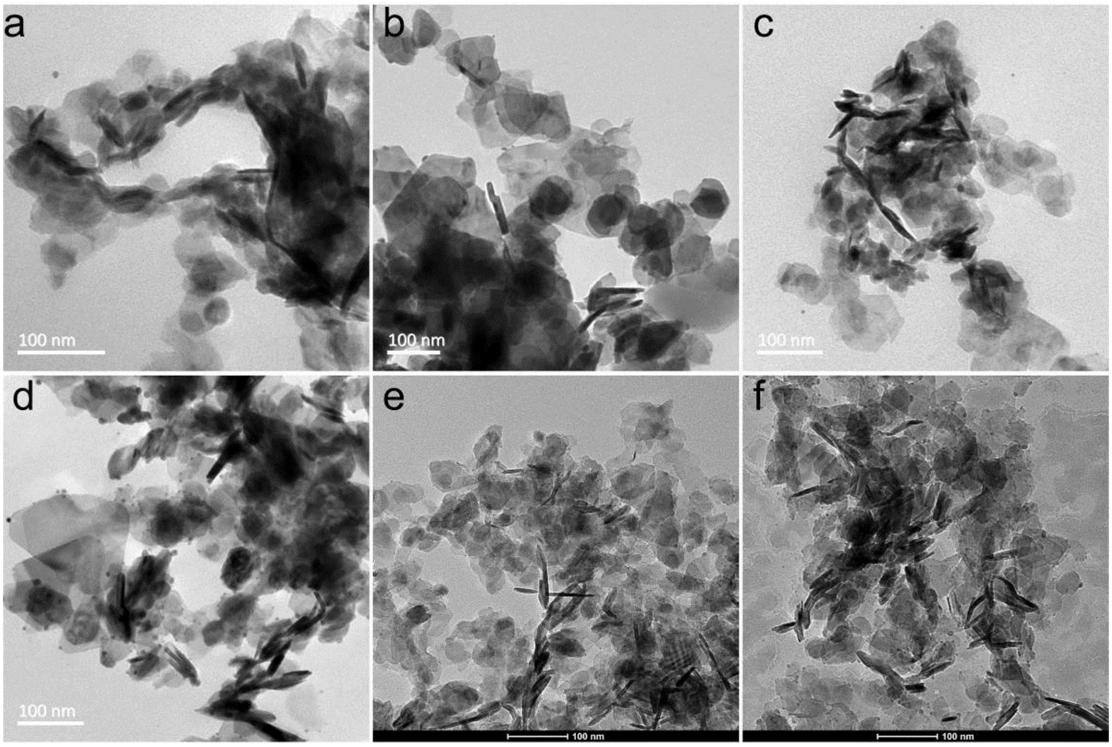

Figure S2. The TEM image of an Ag@MoS_2 nanosheets with Ag content ratios of 1.5 % (a), 2.15 % (b), 2.44 % (c), 2.56 % (d), 3.26 % (e), and 6.72 % (f).

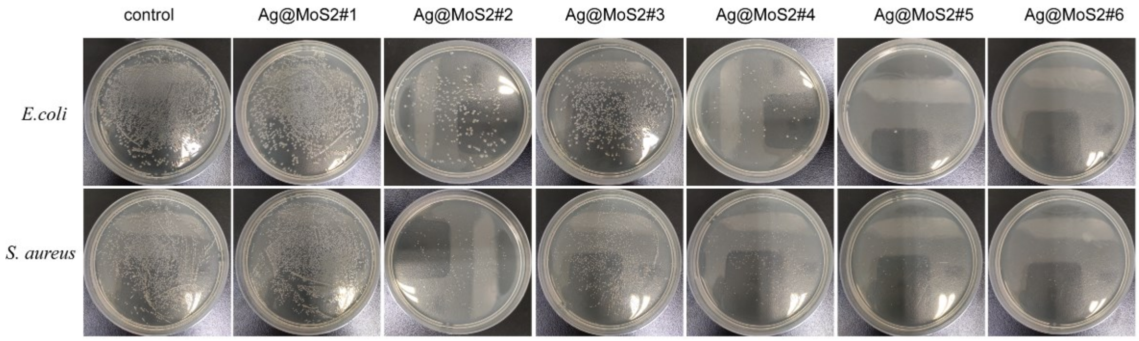

Figure S3. The optical images of *E. coli, S. aureus* incubated 4h with Ag@MoS_2 of 5μg/mL

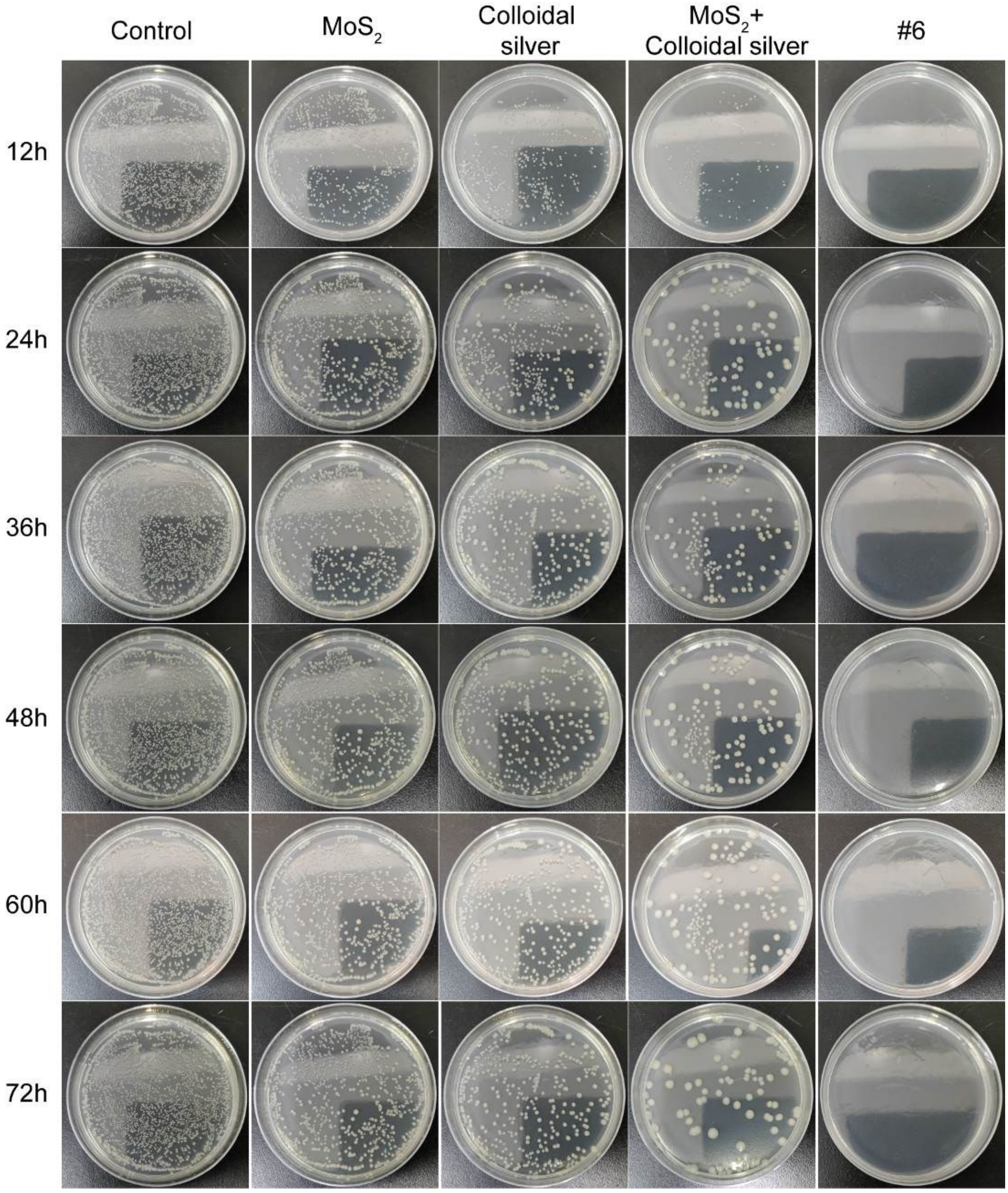

Figure S4. The optical images of the long-term antibacterial effect of samples including #6 (Ag@MoS_2#6), MoS_2, colloidal silver and MoS_2 mixed with colloidal silver.

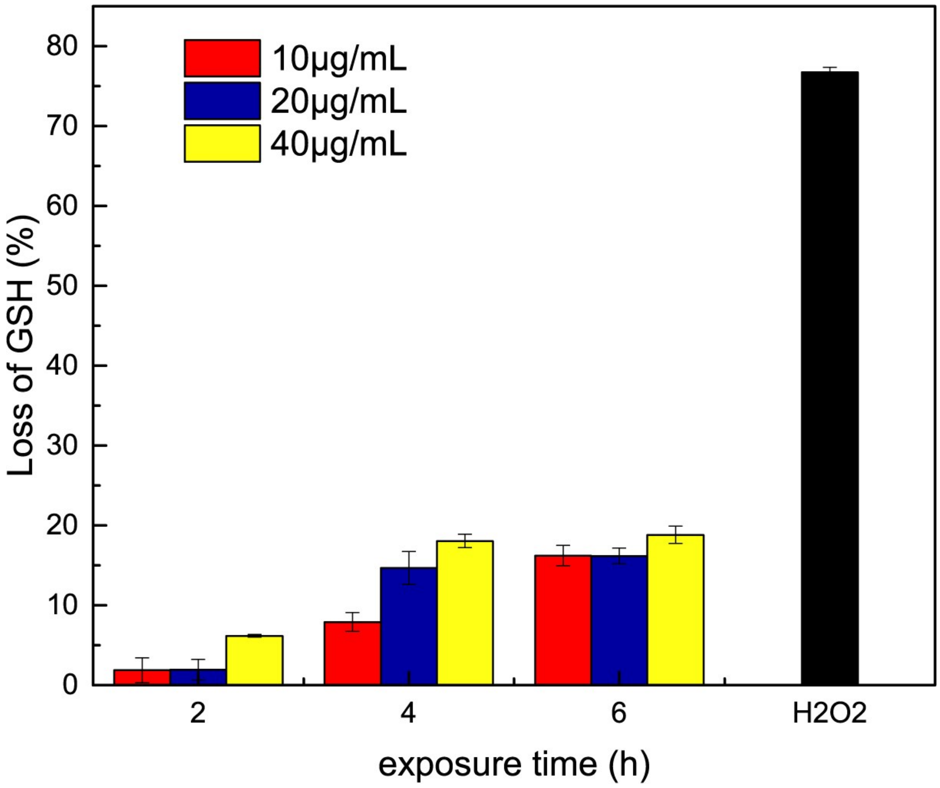

Figure S5. Oxidation of glutathione by Ag@ MoS_2#6. Loss of GSH (0.4 mM) after in vitro incubation Ag@ MoS_2#6 dispersions with different concentrations for 2, 4 and 6h. H_2O_2 (1 mM) was used as a positive control. The bicarbonate buffer without MoS_2 materials was used as a negative control.

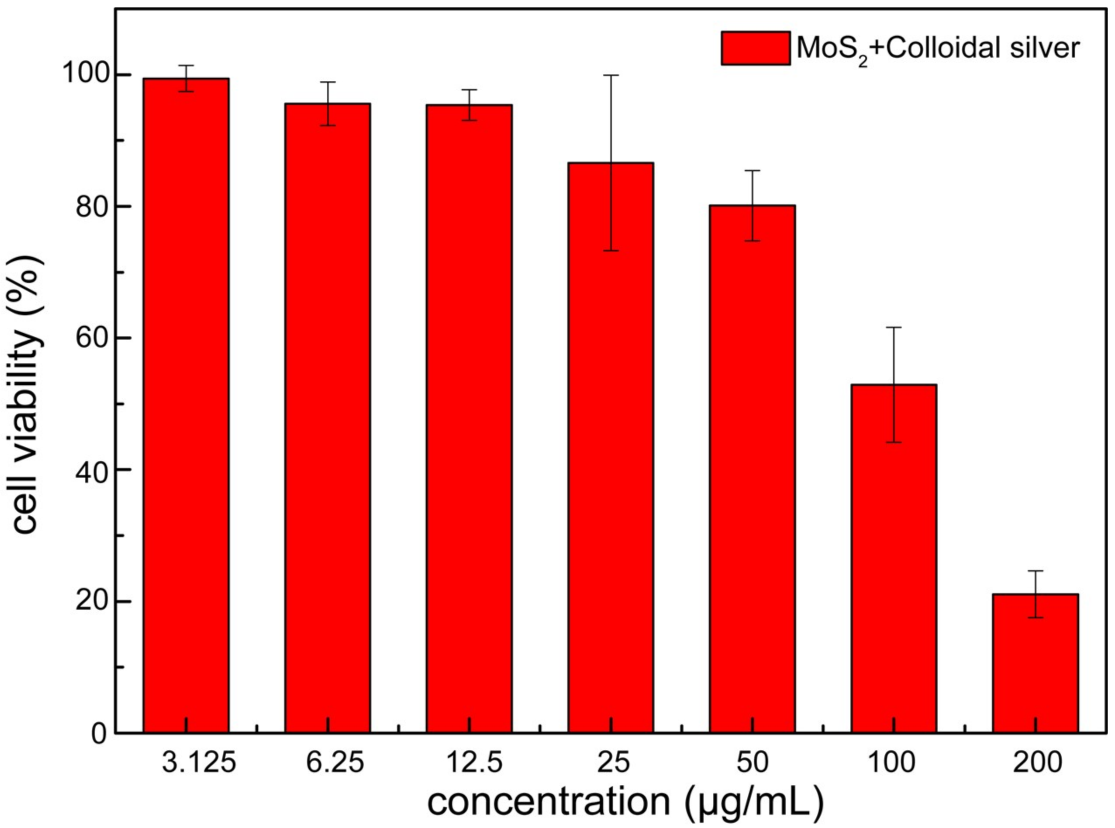

Figure S6. The cytotoxicity of MoS_2 mixed with colloidal silver on BEAS-2B cells. Error bars represented standard deviations (n = 3).
